# Orthodontic Appliance‐Related Mucosal Ulcerations in Newborns and Infants With Craniofacial Disorders

**DOI:** 10.1002/cre2.70291

**Published:** 2026-01-20

**Authors:** Christina Weismann, Kathrin Heise, Katharina Peters, Marit Bockstedte, Matthias C. Schulz, Cornelia Wiechers, Mirja Quante, Christian F. Poets, Bernd Koos, Maite Aretxabaleta

**Affiliations:** ^1^ Department of Orthodontics University Hospital Tuebingen Tuebingen Germany; ^2^ Centre for Cleft Lip, Palate and Craniofacial Malformations University Hospital Tübingen Tübingen Germany; ^3^ Department of Oral and Maxillofacial Surgery University Hospital Tübingen Tübingen Germany; ^4^ Department of Neonatology University Hospital Tuebingen Tuebingen Germany

**Keywords:** cleft lip and/or palate, Robin sequence, Trisomy 21, Tübingen palatal plate

## Abstract

**Objectives:**

In craniofacial disorders (CD) like cleft lip and/or palate (CL/P), Robin sequence (RS) or Down syndrome (DS), an early orthodontic intervention with different palatal plate devices is often applied. However, there are no data on complications such as mucosal ulcerations (MU).To determine the frequency and location of MU and evaluate associations with potential risk factors such as CD type, sex, appliance type, cleft location, underlying syndrome, and anatomical cleft morphology.

**Materials and Methods:**

In a retrospective analysis, we searched our electronic patient records of newborn patients with CD admitted 01/2020‐12/2022 for documented MU. For comparisons, Pearson's Chi‐square or Fisher's Exact test were used, statistical significance was set at *p* < 0.05.

**Results:**

A total of 248 patients (132 female, age range: 0–580 days) receiving 510 palatal plates were treated (51% CL/P, 26% RS; 24% DS); 81/248 (33%) infants had MU, occurring with 112/510 appliances (22%). MU were most common in RS patients (61/69; 88%), followed by CL/P (21/111; 19%). No MU were found in DS. MU occurrence differed significantly by type of orthodontic palatal plate (*p* < 0.0001). There was no significant association between sex, cleft location, presence of an underlying syndrome or cleft morphology and the occurrence of MU.

**Conclusions:**

MU are common during palatal plate therapy. Our findings suggest that MU are correlated with both, the device's function and the mucosal load it applies. Parental training in device use and oral inspection, along with regular clinical check‐ups, is advised to identify MU early, increase appliance quality and patient comfort.

## 1 Introduction

**Figure 1:**
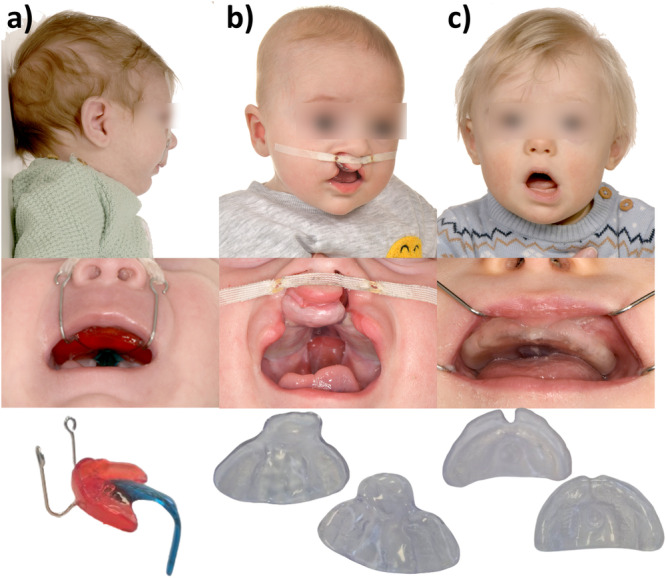
Patients with different craniofacial disorders and their respective palatal plate treatment. For each condition, the figure presents three views: The patient (top), an intraoral photograph of the patient wearing the device (middle) and the respective palatal plate itself (bottom). (a) Patient with Robin sequence with the Tübingen palatal plate. (b) Patient with bilateral cleft lip and patient with palate with a cleft‐covering plate. (c) Down syndrome with a stimulation plate.

In craniofacial disorders (CD), early orthodontic rehabilitation therapy typically starts immediately after birth. This therapy employs various palatal plate devices, each adapted to the specific CD. Palatal plates primarily address three main clinical presentations (Figure 1): Robin sequence (RS) (Wiechers, Arand, et al. 2021), cleft lip and/or palate (CL/P) (Weismann et al. 2024) and Down syndrome (DS) (Castillo‐Morales 1982; Xepapadeas et al. 2020a).

CL/P is caused by a fusion deficiency of the lateral maxillary segments (Cox 2004; Murray 2002), with a prevalence of 0.7:1000 births in Europe (Pérez‐Hettinga et al. 2025). Presurgical palatal plates (Figure 1b) separate the oral from the nasal cavity, promote proper tongue positioning and facilitate sucking, swallowing and drinking (Berkowitz 2006). They also aim to induce an orthopedic effect by guiding and harnessing intrinsic maxillary growth and improving feeding competence (Weismann et al. 2024).

RS is characterized by mandibular retrognathia and glossoptosis, which results in feeding problems, upper airway obstruction (UAO) and in 80%–90% a cleft palate (Robin 1994). It has a prevalence of 1:8.000 births (Santoro et al. 2021). A non‐surgical treatment option to improve UAO and feeding problems is the Tübingen Palatal Palate (TPP) (Figure 1a), a functional orthodontic appliance which is similarly effective as mandibular distraction osteogenesis, yet only minimally invasive (Buchenau et al. 2007; Wiechers et al. 2019; Poets et al. 2017; Effert, Uhlig, et al. 2023; Resnick et al. 2024). It features a velopharyngeal extension which ends just above the epiglottis, shifting the base of the tongue forward to open the airway. To counterbalance the pressure exerted by the tongue on the extension, two extraoral fixation bows are employed. Furthermore, strengthening the extension through a safety wire is recommended (Aretxabaleta et al. 2025). The TPP has a functional effect combined with orofacial stimulation training to enhance mandibular growth (Wiechers, Arand, et al. 2021; Wiechers et al. 2019, 2024 Effert, Uhlig, et al. 2023; Effert, Wiechers, et al. 2023; Wiechers, Iffländer, et al. 2021; Poets et al. 2019).

DS is a chromosomal disorder with a life birth prevalence of 1:1000 (de Graaf et al. 2021). Its phenotype includes generalized hypotonia, orofacial muscle imbalance, brachycephaly, maxillary retrognathia, and a small mouth with a protruding tongue resting on the lower lip. Early orofacial stimulation therapy, based on the Castillo‐Morales concept including the use of a stimulation plate (Figure 1c), is recommended (Castillo‐Morales 1982, 1985; Limbrock et al. 1993, 1991). These plates include a posterior stimulation element to encourage proper tongue positioning and strengthening, thereby reducing oromuscular hypotonia (Bäckman et al. 2003, 2007; Carlstedt et al. 1996, 2001).

Historically, devices were manufactured using a conventional workflow, but a recent shift to a digital approach has improved patient safety, cost and efficiency (Xepapadeas et al. 2020a, 2020b; Weismann et al. 2024; Weise et al. 2022; Aretxabaleta et al. 2025. This transition has increased standardization in device manufacturing, emphasizing accuracy and material safety (Aretxabaleta, Unkovskiy et al. 2021; Aretxabaleta, Xepapadeas et al. 2021) while increasing manufacturing precision. To evaluate device fit, regular follow‐up appointments are essential. During these, oral mucosal lesions (OML), such as mucosal ulcerations (MU) are documented.

This study aimed to determine the frequency and location of MU during palatal plate therapy applied for different CDs. The null hypotheses were:

- There is no difference in the risk of developing MU between different types of CD‐specific palatal plates.
- There is no difference in MU occurrence based on sex, cleft location, or syndromic status in RS patients.

## 2 Materials and Methods

This retrospective, cross‐sectional analysis used data from electronic patient records of the Department of Orthodontics at Tübingen University Hospital, Germany and was approved by the local institutional ethics committee (registration number: 455/2019BO2).

### 2.1 Participants

The clinical electronic documentation of all patients treated at our center between 01/2020 and 12/2022 was reviewed. Inclusion criteria were treatment with an orthodontic palatal plate at our Departments of Orthodontics and Neonatology, respectively, and presence of a CD such as CL/P, RS (isolated or syndromic), or DS. Patients with a CD not receiving an orthodontic appliance were excluded.

### 2.2 Palatal Plate Therapy in Newborns With Craniofacial Disorders—The Interdisciplinary Tübingen Approach

Table 1 contains a short synopsis of the interdisciplinary palatal plate therapy that is applied in Tübingen to treat each CD described in this study. Due to the physiological maxillary growth each patient received routinely more than one palatal plate appliance.

**Table 1:** Description and therapeutic steps of palatal plate treatment at Tübingen for each craniofacial disorder, including the specific devices used.

Craniofacial disorder	Cleft lip and/or palate	Robin sequence	Down syndrome
Appliance type	Presurgical palatal plate	Tübingen palatal plate	Stimulation plate
Therapy start	Directly after birth	Directly after birth	At 3 months of age
Timeframe	Until cleft surgery (18 months of age)	Up to 6–9 months of age	Until eruption of deciduous molars
Control intervals	Every 6–8 weeks	Every 6–8 weeks	Every 8–10 weeks
Appliance renewal	Every 3–4 months	After 3–4 months of therapy	Every 5–6 months
Side‐effects (apart from MU)	Rare skin irritation due to lip tape	Indentations—often observed	Non
Duration of wear	24 h/day (except during cleaning)	24 h/day (except during cleaning)	Intermittently to maintain interest (~1–4 h/day) and during logopedic therapy sessions

The therapy of CL/P patients followed the interdisciplinary Tübingen concept (Weismann et al. 2024). Due to their medical conditions, patients were usually admitted to the Department of Neonatology and received their pre‐surgical palatal plate immediately after birth. In some cases, for example when the patient was born in another hospital with a different treatment approach, patients received their first device as an outpatient in the Department of Orthodontics. Devices were manufactured by a completely digital workflow within 1 day (Weismann et al. 2024). Usually in late morning, an intraoral scan was performed, and the device fitted in the afternoon. The appliance's fit was checked by the orthodontist and parents were trained in handling and cleaning the plate by neonatal nurses. In addition to the plate, extraoral taping is performed with the goal of repositioning the flared cleft segments into a physiological position and to support drinking and swallowing (Abd El‐Ghafour et al. 2020). Both, palatal plate therapy and lip taping should guide growth effectively (Figure 1b). During this initial hospital stay, infants were seen daily in an interdisciplinary visit of orthodontists, neonatologists, nurses, and parents. The appliance's fit was reviewed and the infant's condition as well as its treatment progress (e.g., feeding behavior) were discussed. After discharge, patients were followed up every 6–8 weeks in the Department of Orthodontics to assess treatment progress, as well as appliance fit. Usually, a new appliance was needed every 3–4 months, which was provided during an outpatient visit.

Interdisciplinary treatment of RS patients followed the Tübingen protocol (Wiechers, Arand, et al. 2021; Poets et al. 2019; Aretxabaleta et al. 2025). It began with an inpatient stay at the Department of Neonatology, where infants receive their first TPP directly after admission, ideally within 2 days of birth. Adjustments of the TPP to the patient's anatomical condition included unsedated fiberoptic nasopharyngoscopy of the upper airway and, once optimal fit was suspected, a cardiorespiratory polygraphy (Wiechers, Arand, et al. 2021). Mean duration of hospital stay in isolated as well as syndromic RS was 2–3 weeks. During this time, RS patients were seen daily at least once by their orthodontist and neonatologist. The TPP was adjusted, if necessary, cleaned and the oral cavity examined for MU or other OML. Breathing and feeding status, as well as further treatment steps, were discussed with parents and nursing staff. The first appointment to check the fit of the device in the Department of Orthodontics was scheduled 6 weeks after discharge. A second TPP was routinely fabricated and fitted after 3–4 months during a 3–5‐day hospital stay, which included a new sleep study. Another outpatient visit to assess the TPP's fit was scheduled 6 weeks after this second discharge. TPP treatment was usually discontinued after approximately 6 months. A follow‐up sleep study was performed 2–3 weeks later to rule out recurrence of UAO. If results were normal, TPP treatment was discontinued, and a stimulation plate was provided to further promote physiological mandibular growth and to support the orofacial regulation therapy accordingly to the Castillo‐Morales concept.

Stimulation plate therapy of DS patients was initiated at 3 months of age, as outlined in the German S2K guideline (Deutsche Gesellschaft für Kinder‐ und Jugendmedizin DGKJ und der beteiligten Fachgesellschaften BuwO 2016). For this purpose, a stimulation plate was manufactured by a completely digital workflow (Xepapadeas et al. 2020a). As with the other patient groups, an intraoral scan was performed in the late morning, and the appliance was delivered in the afternoon. This process took place in the Department of Orthodontics with no need for an inpatient stay. Follow‐up visits including check‐ups of the device were performed every 8–10 weeks. A new stimulation plate was typically required every 5–6 months, reflecting the slower facial growth of these patients.

In all CD types, plate therapy was generally discontinued upon eruption of the deciduous molars.

### 2.3 Tissue Examination and Mucosal Ulcerations

**Figure 2:**
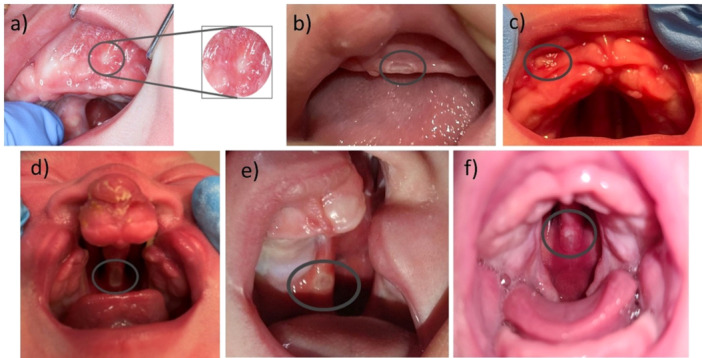
Mucosal ulcerations during palatal plate therapy: (a) Patient with Robin sequence and a cleft palate, exhibiting an oval‐shaped white mucosal ulceration in the left vestibule fold; (b) a lentiform, white mucosal ulceration in its’ initial state at the left side of the maxilla lateral of the labial frenulum; (c) a mucosal ulceration at the right sided mucosal vestibule, being white, lentiform with a small red fringe in a patient with Robin sequence, (d) mucosal ulceration in the posterior vomer region in a patient with a bilateral cleft lip and palate; (e) large mucosal ulceration on the vomer in a patient with a left‐sided cleft lip and palate; (f) patient with Robin sequence and a cleft palate showing a mucosal ulceration in the posterior vomer region.

**Figure 3:**
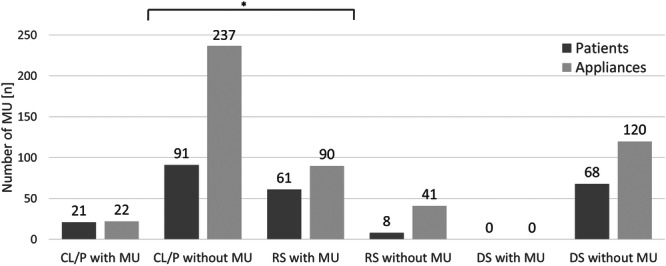
Number (n) of mucosal ulcerations (MU) by patient groups (dark gray) and appliance types (light gray) in cleft lip and/or palate (CL/P), Robin sequence (RS), and Down syndrome (DS). * Indicates a significant difference between CL/P and RS groups by Pearson's Chi‐squared test (*p* < 0.05).

The soft tissue examination was performed by using a small light pen and a tongue depressor during routine clinical check‐ups. MU (so‐called “pressure marks”) were documented in the clinical patient notes. They initially appear as white and lentiform lesions with a red fringe (Figure 2a,b), progressing to an erythema if left untreated (Figure 2c) (Weismann et al. 2024; Miller 1973). Clefting and thin mucosal conditions increase the likelihood of MU, particularly in the vomer region, where many MU already develop in utero (Figure 2d–f), due to the malposition and pressure of the tongue. They may be associated with clinical signs of inflammation (Figure 3d,e). Such lesions must be addressed before continuing treatment, often through chairside grinding and polishing of the appliance. This relieves pressure in the affected mucosal areas, allowing lesions to heal (Weismann et al. 2024). In rare cases, the fabrication of a new plate is required (Weismann et al. 2024).

### 2.4 Statistical Data Analyses

Pseudonymized patient data from electronic patient records were exported to Excel (Microsoft Inc., Redmond, Washington, USA). Statistical analysis and descriptive statistics were performed using JMP (Version 15.2.0, SAS Institute Inc., Cary, NC, USA). Descriptive statistics included frequency, mean, minimum and maximum for each group. Pearson's Chi‐squared test and Fisher's exact test were used to compare categorical variables, with Fisher's test applied when expected cell counts were smaller than 5. A significance level of *α* = 0.05 was used for all statistical tests. In some instances, results were interpreted solely by frequency, without assessing statistical significance.

## 3 Results

### 3.1 Descriptive Analysis

Over a 3‐year period, 253 infants were treated at the Department of Orthodontics, of whom 248 met inclusion criteria; five patients were excluded since they did not receive an orthodontic appliance. The final sample was divided into three groups: 111 CL/P, 69 RS, and 68 DS patients (Table 2). In total, 253 patients were treated with 510 palatal plate devices. Here, CL/P patients received the largest number of appliances (259 plate devices), followed by those for patients with RS (131 plates) and DS (120 plates). The mean age at time of evaluation was 128 days for CL/P (range 0–580), 80 days for RS (range 0–237), and 241 days for DS (range 34–572). A total of 47% were female and most RS patients (84%) had a non‐syndromic, that is, isolated RS. Patients in RS group received minimum 1 and maximum 3 devices during treatment period, DS group 1–5 orthodontic plates and CL/P group needed 1–8 appliances.

**Table 2:** Patient characteristics (number [*n*] and percentage [%]) of the patient groups cleft lip and/or palate (CL/P), Robin sequence (RS) and Down syndrome (DS), respective appliances, sex (female [F], male [M]), for CL/P anatomical cleft morphology (unilateral left or right, bilateral, cleft palate only) and number of syndromic or isolated RS.

	Patient info											
	Total		F		M		Appliances		Age (day)			
	*n*	%	*n*	%	*n*	%	*n*	%	Mean	Median	SD	Min–max
CL/P	111	45	42	38	69	62	259	51	128	108	123	0–580
* Unilateral*	70	63					177					
* Left*	45	64					114					
* Right*	25	36					63					
* Bilateral*	20	18					48					
* Cleft palate*	21	19					34					
RS	69	28	34	49	35	51	131	26	80	76	62	0–237
* Isolated*	110	84					110	84				
* Syndromic*	21						21					
DS	68	27	40	59	28	41	120	24	241	224	135	34–572
*Total*	248	100	116	47	132	53	510	100				

*Note:* Age is shown for each group in days as mean, median, standard deviation (SD, and minimum [min] to maximum [max]) and includes infants who developed MU after fitting their 2nd or 3rd plate.

### 3.2 Incidence of Mucosal Ulcerations

In total, 81/248 (33%) infants developed MU, which were associated with 112/510 (22%) appliances. MU were most common in RS patients, affecting 61/69 (88%) of infants and 90/131 (67%) of TPP devices. In contrast, MU occurred in 21/111 (19%) of CL/P patients with 22/259 (8%) palatal plate appliances, while no MU were observed in DS patients (Figure 3).

The type of CD—and consequently the use of orthodontic palatal plates—significantly influenced the occurrence of MU (*p* < 0.0001) (Table 3). Over the 36‐months (1096 days) observation period, RS devices required adjustments on a total of 215 days, more than the 23 days for CL/P devices. Nearly all CL/P‐MU healed after one single adjustment, except one, while 48 lesions from RS devices needed multiple adjustments. Most devices caused MU in more than one intraoral location—this was true for 99% of RS devices and 96% of CL/P devices. MU occurred in 78% of the first TPP, compared to 54% of patients who had their second TPP. MU appeared between 1 and 16 days (mean time 3) after insertion of a new TPP device, while it took between 1 and 30 days (mean time 5) until they had healed without further complications.

**Table 3:** Statistical analysis of differences in the occurrence of mucosal ulcerations (MU) among patient groups with cleft lip and/or palate (CL/P), Robin sequence (RS), and Down syndrome (DS).

Variables	CL/P				RS		DS	Test	*χ* ^2^	*p*‐value
**Type of palatal plate**	22/259				90/131		0/120	*χ* ^2^ (df=2)	228.18	< 0.001*
	22/259				90/131		—	*χ* ^2^ (df=1)	154.06	< 0.001*
**Cleft morphology**	**L**	**R**	**B**	**P**						
	13/114	6/63	3/48	0/34	—		—	Fisher	/	0.1596
**Syndrome‐association**					**Isolated**	**Syndromic**				
	—				79/110	11/21	—	*χ* ^2^ (df=1)	3.10	0.0784
**Sex (per group)**	F: 6/101				—		—	*χ* ^2^ (df=1)	1.10	0.2934
	M: 16/158									
	—				F: 44/60		—	*χ* ^2^ (df=1)	1.39	0.2386
					M: 46/71					
**Sex (total)**	F: 50/234							*χ* ^2^ (df=1)	0.09	0.7675
	M:62/276									

*Note:* The number of mucosal ulcerations is expressed as number of MU/total. Variables analyzed include type of palatal plate, sex (female [F], male [M]), anatomical cleft morphology (left [L], right [R], bilateral [B], and cleft palate only [P]), and syndrome association (isolated vs. syndromic) within the RS group. The applied statistical tests are Pearson's chi‐squared test (*χ*²) with degrees of freedom (df) or Fisher's exact test, as indicated, along with corresponding *p*‐values. Statistically significant results (*p* < 0.05) are denoted as (*).

MU were most common in unilateral cleft formations (19/61 [31%]), in detail in 13/61 (21%) left‐sided CL/P patients, followed by right‐sided clefts (6/61 [10%]) and bilateral cleft locations (3/61 [5%]). No MU was related to the use of palatal plates in patients with an isolated cleft palate. The anatomical cleft morphology in the CL/P group had no significant influence on the frequency of a MU (Table 3). Among RS patients, 11/21 (52%) with syndromic and 79/110 (72%) with non‐syndromic RS had a MU. However, the association with a syndrome had no significant influence on developing a MU (Table 3). There was no statistically significant difference between male and female in the risk of developing MU (Table 3).

### 3.3 Anatomical Area Affected by a MU Among CL/P and RS Patients

In CL/P patients, the most frequently affected area was the posterior vestibule (5/61 appliances, 8%) followed by the alveolar ridge (4/61 appliances, 5%) and the soft palate (3/61 appliances, 5%) (Table S1). RS patients showed a larger amount of affected anatomical areas compared to CL/P. The most frequent areas were the vestibule (isolated in 31/90 TPP devices, 31%), often in combination with the lip frenulum (13/90 appliances, 14%) or the maxillary tuberosity region (10/90 appliances, 11%). An isolated tuberosity region MU was observed in 8/90 TPP appliances (9%).

## 4 Discussion

The aim of this study was to assess clinical side effects of orthodontic palatal plates in newborns and young infants with CD, with particular emphasis on the incidence, risk factors for and localization of MU. To our knowledge, this represents the first such comprehensive evaluation.

The types of CD and appliance were significantly associated with MU occurrence, leading to the rejection of the first null hypothesis. This association may be explained by the specific function of the TPP in infants with RS. MU occurred in 88% of infants with RS during therapy, consistent with the expected forces involved. MU were observed in 19% of infants with CL/P and in none of the patients with DS. In RS patients, the palatal base plate and extraoral fixation bows of the TPP aim to counterbalance the pressure exerted by the extension on the tongue (Aretxabaleta et al. 2025). For CL/P patients, the plate guides lateral segment growth to reduce cleft width (Weismann et al. 2024). Both, TPP and CL/P palatal plate exert a constant force and are worn continuously, whereas plates for infants with DS are worn only intermittently (e.g., 2 h/day for 6 days/week). Unlike RS and CL/P plates, stimulation plates are not needed for feeding, which introduces additional stress and forces on the mucosa in RS and CL/P devices. The authors hypothesize that devices exposed to greater forces and physiological movements are more likely to exert mucosal strain, potentially causing MU.

Further, the manufacturing workflow should also be considered. While previous accuracy studies showed no clinically relevant difference between conventionally and digitally manufactured plates (Aretxabaleta, Unkovskiy et al. 2021a), challenges in data acquisition—such as artefacts or missing maxillary data—may contribute to the formation of MU (Weismann et al. 2024). Poor maxillary scan quality may increase the likelihood of MU in specific areas. Scanning is more difficult in RS and CL/P patients due to anatomical complexities, like restricted mouth opening in RS and the extent of the cleft defect in CL/P (Weismann et al. 2024; Weise et al. 2022). In contrast, DS patients, who begin therapy around 3 months of age, are easier to scan, reducing the risk of MU. Additionally, manufacturing of a conventional TPP involves the additive manufacturing of a maxillary model, which serves as the basis for the final appliance. In contrast, devices for CL/P and DS are completely digitally designed and directly additively manufactured, omitting this intermediate step. This additional step in the TPP workflow may introduce variability and could also help explain the higher incidence of MU associated with the TPP.

The second null hypothesis can be accepted, as no statistically significant differences in MU occurrence were found between cleft morphology, the presence of a syndrome in RS patients, or sexes. Therefore, the authors conclude that the medical conditions in our patients had no role in the pathogenesis of MU. Due to a lack of literature on side effects of orthodontic appliances in newborns with CD, comparisons can only be made with complete dentures in adult edentulous patients. One study examined the frequency of MU in 60 complete denture patients, who were assessed from 1 day after appliance placement until achieving patient comfort. Adjustments were needed in 86% of patients (Sadr et al. 2011), which is comparable to our results in RS patient. This suggests that the strain experienced by the TPP may be similar to that seen with complete dentures. The study noted no significant sex differences among patients with dentures, which aligns with our findings.

The anatomical regions mainly affected by MU varied between CL/P and RS patients. These findings highlight the need for both technicians and clinicians to focus on these areas during the manufacturing process. First, the intraoral scan should be carefully evaluated for quality and completeness (Weise et al. 2022). In RS patients, one of the areas most prone to scan‐related MU is the posterior region like the maxillary tuberosity, likely due to restricted mouth opening (Weise et al. 2022). As previously discussed, incomplete scans can lead to ill‐fitting appliances and subsequent MU. The vestibule was the most frequently affected area (13% of all MU). This is likely due to the challenge of capturing the transition from fixed to movable gingiva, the scanner head's size, potentially leading to overextended flanges on the appliance. Another common area for MU was the anterior lip frenulum, likely due to the TPP's role in counterbalancing tongue pressure. Technicians should pay close attention to these regions to prevent overextension, particularly in the vestibule and posterior area. A thorough quality check is crucial before clinical use (Aretxabaleta et al. 2025). Sadr et al. evaluated maxillary injuries with dentures, with the posterior palatal seal area of the soft palate (27%) being the most frequently injured location, followed by the buccal slope of the residual ridge (14%), the distobuccal sulcus (13%), and the labial frenulum (10%). In contrast, the least affected areas were the hard palate and mid‐palatal suture (0%), incisive papilla (0.6%), maxillary tuberosity (3%), and buccal and labial sulci (5%) (Sadr et al. 2011). This differs from our data with the TPP, where the vestibule was affected most often (34%), followed by the vestibule in combination with the lip frenulum (14%) and the tuberosity (9%). Both appliances exert significant stress on the mucosa, but in different regions, which is associated with the occurrence of MU. Notably, the tuberosity area acts as a hypomochlion in the biomechanical function of the TPP, serving as the pivot point or abutment of a lever for the velopharyngeal extension, which enhances the leverage effect exerted by the tongue. Therefore, this area experiences increased loading, especially during jaw movements, for example swallowing. With increased speech therapy, an increase in MU can also be anticipated.

Budtz‐Jørgensen (1981), also studying OML associated with removable dentures in prosthetic patients, also found that the primary etiological factor for MU was the overextension of denture borders. This principle also applies to plate therapy, highlighting the critical importance of device fabrication, as well as the need for consistent quality checks throughout this treatment.

Every 14 to 21 days, oral epithelial cells are replaced through cell division to match the high functional demands placed on the oral mucosa (Brizuela and Winters 2024). The facial area has an increased blood circulation compared to the rest of the body (Hayashi et al. 2021), which enhances its healing capacity to reduce long‐term consequences (Weismann et al. 2024). The oral epithelium is highly resistant and protective, which benefits palatal plate therapy (Groeger and Meyle 2019). Throughout the study, no long‐term adverse reactions were documented and the MU healed without consequences during an average period of 5 days. The vestibule, lip frenulum and soft palate were the most frequently affected regions for MU in both patient groups. Consequently, it is advisable to provide ample space for the orthodontic appliance to avoid lesions in these movable anatomical areas, allowing for adequate movement without compromising the mucosa.

It is essential to emphasize the importance of training caregivers in the proper use of the device to prevent malalignment and conduct daily routine inspections of the oral mucosa. This practice helps to identify irregularities and alerts the clinician to prevent further issues. Additionally, regular outpatient appointments facilitate early detection of MU and allow for timely adjustments to accommodate patient growth and enhance comfort, although we wish to stress that the mucosa seems to adapt to the plates, as most MU occur in the first 5–10 days of treatment. These factors may be responsible for our observation that MU only rarely continue to arise after discharge.

### 4.1 Limitations and Outlook

Several limitations should be noted. This study is based on retrospective data from a single center. Additionally, for ethical reasons, it is impossible to determine whether the incidence of MU would be lower with a conventional than with a digital workflow. Furthermore, potential bias may stem from correlating devices with different functions, as no side effects were reported for stimulation plates.

Due to the lack of appropriate literature, comparisons with other cleft centers and patient cohorts using palatal plates were not possible. Future studies are essential to raise awareness, improve therapy practices for orthodontic palatal plates, and enhance patient comfort.

### 4.2 Conclusion

The occurrence of MU differed significantly across CD. Palatal plates are subject to considerable forces during physiological movements, which are likely to strain the mucosa and cause MU. A total of 88% of RS group infants developed MU, compared to 0% in the DS group, suggesting that the amount of strain exerted and duration of daily use are relevant factors. No associations were found between MU occurrence and sex, cleft location, or the presence of an associated syndrome in RS patients. A quality check of the device is essential before clinical use. Movable anatomical areas such as the vestibule, lip frenulum and soft palate should be adequately spared in the appliance to allow for proper physiological movements. Additionally, special attention should be given to regions such as the maxillary tuberosity, the alveolar ridge and the edge of the cleft as these are more prone to MU. Parental training in device handling and oral mucosal inspection, alongside regular clinical appointments, are recommended for the early management of MU, preventing aggravation, increasing appliance quality and patient comfort.

## Author Contributions

Conceptualization: Maite Aretxabaleta, Bernd Koos, and Christina Weismann. Data curation: Kathrin Heise, Cornelia Wiechers, Mirja Quante, Katharina Peters, Maite Aretxabaleta, and Christina Weismann. Formal analysis: Marit Bockstedte, Matthias C. Schulz, Maite Aretxabaleta, and Christina Weismann. Investigation: Kathrin Heise, Marit Bockstedte, Maite Aretxabaleta, and Christina Weismann. Methodology: Matthias C. Schulz, Bernd Koos, Marit Bockstedte, Maite Aretxabaleta, and Christina Weismann. Visualization: Marit Bockstedte, Maite Aretxabaleta, and Christina Weismann. Writing—original draft: Maite Aretxabaleta and Christina Weismann. Writing—review and editing: Marit Bockstedte, Maite Aretxabaleta, and Christina Weismann, Katharina Peters, Christian F. Poets, Cornelia Wiechers, Mirja Quante, Matthias C. Schulz, and Bernd Koos. All authors agree to be accountable for all aspects of the work. All authors critically revised the manuscript and gave their final approval for publication.

## Funding

The authors received no specific funding for this work.

## Ethics Statement

The study was conducted in accordance with the Declaration of Helsinki, and approved by the Institutional Review Board (or Ethics Committee) of Tuebingen University hospital (protocol code 455/2019BO2 and date of approval 20 March 2025).

## Consent

Patient consent was waived following the current version of §45 (3) 4 and §46/2a of the Federal State Hospital Act of Baden Württemberg that specifies that the use of patient data of the hospitals’ own patients does not require the informed consent of the patients or their legal guardians (Dietz O. Landeskrankenhausgesetz Baden‐Württemberg (LKHG): Kommentar. Wiesbaden: Kommunal‐ u. Schul‐Verl. Heinig; 1988).

## Conflicts of Interest

The authors declare no conflicts of interest.

## Supporting information


**Table S1:** Anatomical area of mucosal ulcerations among cleft lip and/or palate (CL/P) and Robin sequence (RS) patients are presented by number (n) and frequency (percentage [%]).

## Data Availability

Data available by the corresponding author upon reasonable request. Information to patients is not available given the patient data protection law.
