# Supplementary material for: Orthodontic Appliance‐Related Mucosal Ulcerations in Newborns and Infants With Craniofacial Disorders
Source: Clin Exp Dent Res. 2026 Jan 20;12(1):e70291. doi: 10.1002/cre2.70291 (PMC12820415; doi:10.1002/cre2.70291)
Supplement: Supplementary file 1 — Table S1: Anatomical area of mucosal ulcerations among cleft lip and/or palate (CL/P) and Robin sequence (RS) patients are presented by number (n) and frequency (percentage [%]). [file CRE2-12-e70291-s001.docx]

| CL/P | | | RS | | |
| --- | --- | --- | --- | --- | --- |
|  | *n* | *%* |  | *n* | *%* |
| Anatomical area |  |  | **Anatomical area** |  |  |
| Vestibule posterior | 5 | 8 | Vestibule | 31 | 34 |
| Alveolar ridge | 4 | 7 | Vestibule and lip frenum | 13 | 14 |
| Edge of the cleft | 3 | 5 | Vestibule, maxillary tuberosity | 10 | 11 |
| Soft palate | 3 | 5 | Maxillary tuberosity | 8 | 9 |
| Incisive papilla | 1 | 2 | Lip frenum | 3 | 3 |
| Premaxilla | 1 | 2 | Vestibule, Edge of the cleft | 3 | 3 |
| Maxillary tuberosity | 1 | 2 | Vestibule, maxillary tuberosity and edge of the cleft | 3 | 3 |
| Soft palate and vestibule | 1 | 2 | Edge of the cleft | 2 | 2 |
| Vestibule anterior | 1 | 2 | Cheek frenum | 2 | 2 |
| Vomer | 1 | 2 | Vestibule, lip frenum and edge of the cleft | 2 | 2 |
| Cheek frenum | 1 | 2 | Vestibule, lip frenum and maxillary tuberosity | 2 | 2 |
|  |  |  | Vestibule, maxillary tuberosity and lip frenum | 1 | 1 |
|  |  |  | Vestibule, vomer | 1 | 1 |
|  |  |  | Vestibule, base of tongue | 1 | 1 |
|  |  |  | Vomer | 1 | 1 |
|  |  |  | Vomer, lip frenum | 1 | 1 |

Table S1: Anatomical area of mucosal ulcerations among cleft lip and/or palate (CL/P) and Robin sequence (RS) patients are presented by number [n] and frequency (percentage [%]).
